# Supplementary material for: Publication authorship: A new approach to the bibliometric study of scientific work and beyond
Source: PLoS One. 2024 Apr 18;19(4):e0297005. doi: 10.1371/journal.pone.0297005 (PMC11025840; doi:10.1371/journal.pone.0297005)
Supplement: S1 Appendix — (PDF) [file pone.0297005.s001.pdf]

Supporting information

S1 Appendix. Top-10 Journals in Accounting, Astronomy, and Gastroenterology.

Table 17. Top-10 Journals in Accounting, Astronomy, and Gastroenterology.

Rank	Journal	Articles
1	Journal of Finance	670
2	Journal of Accounting Research	316
3	Journal of Financial Economics	1227
4	Review of Financial Studies	784
5	Management Accounting Research	193
6	Journal of Technology Transfer	543
7	Accounting Review	577
8	Journal of Accounting and Economics	389
9	British Accounting Review	272
10	Accounting, Organizations and Society	362
	Total Articles in Accounting Journals	5333
1	Annual Review of Astronomy and Astrophysics	37
2	Astronomy and Astrophysics Review	19
3	Living Reviews in Solar Physics	34
4	Annual Review of Earth and Planetary Sciences	145
5	Space Science Reviews	219
6	Astrophysical Journal Letters	6402
7	Astrophysical Journal, Supplement Series	1688
8	Publications of the Astronomical Society of Australia	368
9	Nature Astronomy	233
10	Monthly Notices of the Royal Astronomical Society: Letters	1672
	Total Articles in Astronomy Journals	10817
1	Gut	1661
2	Nature Reviews Gastroenterology and Hepatology	50
3	Gastroenterology	2594
4	The Lancet Gastroenterology and Hepatology	164
5	Alimentary Pharmacology and Therapeutics	1841
6	Gut Microbes	394
7	Journal of Crohn's and Colitis	1186
8	Gastric Cancer	866
9	Journal of Gastroenterology	1086
10	American Journal of Gastroenterology	1451
	Total Articles in Gastroenterology Journals	11293
