# Supplementary material for: Publication authorship: A new approach to the bibliometric study of scientific work and beyond
Source: PLoS One. 2024 Apr 18;19(4):e0297005. doi: 10.1371/journal.pone.0297005 (PMC11025840; doi:10.1371/journal.pone.0297005)
Supplement: S2 Appendix — (PDF) [file pone.0297005.s002.pdf]

S2 Appendix. Glossary.

Assortativity. Probability that a vertex connects to similar vertices. Most common similarity is the degree of a vertex. Provides an idea of the neighborhood connectivity of vertices.

Author bibliographic coupling analysis. Bibliometric method that measures the similarity and relationship between authors based on their shared references. It can reveal the social structure and evolution of a research field by identifying clusters of authors who share common themes, topics, or methods.

Author co-citation analysis. Bibliometric method that measures the similarity and relationship between authors based on how often they are cited together by other authors. It can reveal the social structure and evolution of a research field by identifying clusters of authors who share common themes, topics, or methods.

Betweenness. Number of shortest paths between vertices that go through a vertex. Provides an idea how central a vertex is in terms of being a gatekeeper or bottleneck between other vertices.

Bibliographic coupling analysis. Bibliometric method that measures the similarity between publications based on the references they have in common. It can reveal the intellectual structure and evolution of a research field by identifying clusters of publications that are related by common themes, topics, or methods.

Bibliometrics. Set of statistical methods to analyze and measure various aspects of scholarly literature and otherwise information sources (patents, laws, etc.). It can reveal the historical development, patterns, trends, impacts, and relationships of subject fields, authors, publications, and citations in order to understand and evaluate the state and evolution of science and knowledge production.

Closeness. Average length of shortest paths from a vertex to all other vertices. Provides an idea of how central a vertex is in terms of how close other vertices are to it.

Clustering coefficient. See *Transitivity.*

Co-authorship analysis. Bibliometric method that measures the collaboration between authors based on their joint publications. It can reveal the social structure and evolution of a research field by identifying clusters of authors who share common themes, topics, or methods.

Co-citation analysis. Bibliometric method that measures the similarity between publications based on their shared citations. It can reveal the intellectual structure and evolution of a research field by identifying clusters of co-cited publications that represent topics or paradigms.

Co-word analysis. Bibliometric method that measures the similarity and relationship between words based on how often they appear together in a collection of documents. It can reveal the intellectual structure and evolution of a research field by identifying clusters of words that describe themes or topics.

Communicative constitution of organization. Theoretical perspective that claims that communication is not something that happens within or between organizations, but rather the very process that creates organizations. It challenges the conventional view of organizations as containers of communication and offers a new way of understanding organizational phenomena.

Degree. Number of edges that connect to a vertex. Provides an idea of how central a vertex is in terms of how well it is immediately connected to other vertices.

Density. Ratio of the number of edges present in a graph and the maximum number of edges the graph can contain. Provides an idea of how densely a graph is connected.

Direct citation. Type of citation analysis that measures the influence of a publication based on the number of times it is cited by other publications. It can be used to assess the impact and quality of a publication, as well as to identify the scientific frontier and core publications.

Edge. Fundamental unit of analysis that together with two vertices forms a graph.

Isolate. A vertex without an edge, disconnected from a graph.

Keyword analysis. Bibliometric method that describes the topic of a publication based on the frequency or distribution of keywords. It is used to study the content and trends of academic areas.

Latent Dirichlet Allocation. Probabilistic model to infer latent topics that occur in a collection of documents such as scientific publications. It assumes that each document is a mixture of topics, and each topic is a distribution over words.

Publication authorship analysis. New bibliometric method that measures the similarity and relationship between publications based on their shared authorship. It can reveal the intellectual structure and evolution of a research field by identifying clusters of publications that are related by common, themes, topics, or methods.

Scientometrics. See *Bibliometrics.*

Transitivity. Probability that adjacent vertices of a vertex are connected. Provides an idea of neighborhood segmentation of vertices.

Vertex. (Plural: vertices). Fundamental unit of analysis that together with an edge forms a graph.
