# Supplementary material for: Publication authorship: A new approach to the bibliometric study of scientific work and beyond
Source: PLoS One. 2024 Apr 18;19(4):e0297005. doi: 10.1371/journal.pone.0297005 (PMC11025840; doi:10.1371/journal.pone.0297005)
Supplement: Attachment — Submitted filename: Response to Reviewers.pdf [file pone.0297005.s003.pdf]

Dear Roy: Thank you for your guidance on how to tackle the issues raised by the reviewers. I have been closely following your suggestions. You will find my responses to the reviewers below.

Reviewer #1

Dear Reviewer #1: Thank you for your comments on my manuscript. In the following, I will take upon each one of them and offer revisions accordingly.

Practical Solution

You start by saying that the "paper lacks sufficient attraction. Using publication authorship as the connection point of the literature coupling network might improve the clustering quality, but the author should first provide a feasible practical solution for other researchers to validate."

The most practical solution I can provide is access to the source code my analysis is based on. Both the code as well as a step-by-step research protocol of how to obtain the data sets I used in my illustrations are available on GitHub (cf. reference on p. 4). The protocol may easily be adapted to curate data sets of your own interest, of course.

Tables

You continue with the observation that "the figures and tables used in the paper do not effectively help readers understand the author's viewpoints. The terms used in the tables such as age, strength, and degree are not clear enough for readers to understand what the meaning is."

I value this observation, but I must ask you to specify what you are looking for. The paper describes common measures in network analysis in layperson's terms (e. g., saying that the degree is the number of edges a vertex has to other vertices, cf. p. 9) as well as points to the appropriate references on how these measures are used in the literature (e. g., Leydesdorff on betweenness as an indicator of interdisciplinarity, cf. p. 9). One issue that came about your observation is that age was not identified with its units. I corrected this shortcoming, and now age is given in years.

Figures

You end on the comment that "the figures are not clear enough."

I agree with you that some figures are of low quality (i. e., they appear blurry). The issue only occurred when the PLOS ONE submission engine stitched together the manuscript and the separately uploaded figures. The figures are high-quality PostScripts to begin with, so I managed a workaround by including them as attachments to the manuscript myself. The issue is thus hopefully resolved.

Reviewer #2

Dear Reviewer #2: Thank you for your comments on my manuscript. In the following, I will take upon each one of them and offer revisions accordingly.

Structure

You say that "the paper is a bit unstructured" and already ask to "introduce [the] research design before going through the analysis of the case study."

I am glad that you bring this issue up. The introduction now includes an overview of the research design. I hope by this you and the readers do not get lost in the technical details but remain on track following the research step by step.

Theoretical Background

"The description of the theoretical background is scarce," you mention. In this connection, you ask that the briefly introduced bibliographic approaches should be discussed in a more extensive way.

I am happy to accommodate this suggestions. The theoretical considerations are now divided into three subsections (on the intellectual structure, the social structure, and the combination of both these structure of scientific work). The considerations fill in details on each one of the five established bibliometric approaches (i.e., co-citation, bibliographic coupling, author co-citation, bibliographic coupling, and co-authorship) including a larger list of references to existing literature. I hope that this satisfies your call for a more broader theoretical background in the sense of a brief literature review. I refrain from going into more detail since in my humble opinion this would unbalance the introductory part of the paper opposite the empirical illustrations and statistical analysis.

Co-word and Topic Networks

You argue that "co-word and topical networks should be considered as well" and readily suggest to compare "the results obtained through publication authorship not only with bibliographic coupling, but also with co-citation, co-word and topical networks."

I fully agree with you that "it would be highly relevant to assess if publication authorship provides similar or different results compared to co-word and topical networks." However, there is a simple reason why I cannot provide a full-fledged analysis of co-citation, co-word, and topic networks alongside bibliographic coupling and publication authorship. PLOS ONE does not explicitly limit the number of pages a paper can have, but it encourages a concise and accessible writing style. My analysis and discussion of bibliographic coupling opposite of publication authorship already make up more than half the paper. I estimate that an analysis of co-citation, co-word, and topic networks would

add at least eight to ten pages. Next to an increase in the length of the paper, its readability would likely suffer from a comparison between publication authorship and now four (instead of one) other bibliometric approaches.

I am therefore not providing a full-fledged analysis of co-citation, co-word, and topic networks. Instead, you will find a more theoretical discussion of bibliometric networks based on word co-occurrences alongside an outlook of further work in the conclusion.

Commonalities and Differences Between Fields of Research

You point out that the analysis of the three academic areas (accounting, astronomy, and gastroenterology) is very detailed, yet it lacks comments on the differences between them. You specifically suggest to look into "specific behaviors in each research field that motivate higher/lower density, transitivity, assortativity, and so on."

Again, thank you for these insightful suggestions, which I gladly take up. Of course, you are absolutely right that each academic discipline has its unique features (e.g., a lower average number of authors on papers in accounting than in gastroenterology, mentioned on p. 5). This then immediately reflect in the findings such as a different densities of bibliometric networks. My empirical illustrations, however, pit bibliographic coupling against publication authorship across disciplines. They are not meant to be comparing academic areas to one another.

To take up your point, I have now included some clarifying remarks at the beginning of the data section. I hope this will satisfy your curiosity of differences between and among fields of research for now.

Glossary

"It could be appropriate to define a sort of 'glossary of network metrics and properties' rather than introducing them one by one within the text," you mention.

Yes, perhaps it is a good idea to include a glossary for the reader. I will consult with the editor in what way he thinks a glossary is appropriate to include.

Tables

You point out "two typos in Table 5 and Table 6. The density values corresponding to publication authorship should be 0.001 and 0.004, respectively."

Thank you for doing so. You are, of course, correct. The reported values should have been and now do read "< 0.01" (because PLOS ONE asks for only two decimal places).
