# Supplementary material for: Publication authorship: A new approach to the bibliometric study of scientific work and beyond
Source: PLoS One. 2024 Apr 18;19(4):e0297005. doi: 10.1371/journal.pone.0297005 (PMC11025840; doi:10.1371/journal.pone.0297005)
Supplement: Attachment — Submitted filename: Response to Reviewers (R2).pdf [file pone.0297005.s004.pdf]

Dear Roy:

Thank you for your guidance on how to tackle the issues raised by the reviewer. I have been closely following your suggestions. You will find my responses to the reviewer below.

I would also like to thank you and the reviewer for your patience with the revision. There is no simple way to explain why it took me so long to properly address your critique other than saying that life sometimes gets in the way. I apologize and certainly hope that you find the revision to your satisfaction.

Reviewer #2

Dear Reviewer #2: Thank you for your extended comments on my manuscript. In the following, I will take upon each one of them and offer revisions accordingly.

Theoretical Background

Following your argument that "literature review on existing works in which the methods are described and applied is essential," I now discuss highly-cited research on the intellectual and social structure of scientific work. While this brief review of existing works is necessarily selective, I also include references to comprehensive reviews of the theory and practice of bibliometric studies. My hope is the examples of previous work and the pointer to reviews furthers my argument that publication authorship is a valid alternative to mainstay methods in bibliometrics.

Topic and Co-word Networks

You "consider it necessary to analyze at least one among topical and co-word networks" in order to compare it to bibliometric coupling and publication authorship. I am happy to report that the manuscript now includes not just one but two alternative methods in the discussion.

First, I ran a co-word analysis on words in the abstracts of articles in accounting, astronomy, and gastroenterology. The results of the analysis are unsurprisingly not as pronounced as the more granular picture that publication authorship delivers, mostly due to the fact that co-word analysis necessarily yields mutually exclusive word clusters.

Second, I ran Latent Dirichlet Allocations (LDAs) for the three academic areas in question. The results are in line with publication authorship if not somewhat more refined as the now included example in accounting shows. However, there are two major drawbacks to running LDAs. On the one hand, they require the researcher to fix the number of clusters a priori, which always entails a particular level of arbitrariness. On the other hand, the underlying machine learning algorithm of LDAs forced me to switch from my personal computer to a high-performance computing platform in the cloud where computations still ran for hours.

I hope that this newly included discussion of alternative methods in bibliometric studies satisfies your curiosity.

Glossary

As per your suggestion, the revised manuscript now includes a glossary of the most prominent concepts.

Additional Refinements

The second revision of the manuscript includes several refinements. Most importantly, an update to one of the R packages I relied on earlier prompted me rewrite my own code. In the process of doing so I had to discover that this previously used package roughly doubles the values for the basic document-term matrix for publication authorship, whereupon inflated values made it into the original reporting of the results.

After double (and triple, and quadruple) checking my own code, I can safely report that all reported values still yield the same implications. (After all, the previously used package merely doubled the values, which does not change much in the relation among numbers.) In the course of action, I have streamlined the discussion of the findings in accounting, astronomy, and gastroenterology. Hopefully the manuscript is now a more straight-forward read, not least because it quicker makes it to the discussion of alternative methods in bibliometric studies.
