# Supplementary material for: Publication authorship: A new approach to the bibliometric study of scientific work and beyond
Source: PLoS One. 2024 Apr 18;19(4):e0297005. doi: 10.1371/journal.pone.0297005 (PMC11025840; doi:10.1371/journal.pone.0297005)
Supplement: Attachment — Submitted filename: Response to Reviewers (R3).pdf [file pone.0297005.s005.pdf]

Dear Roy, dear Reviewer #2:

Again, thank you for your comments on my manuscript. I have happily taken up your suggestions:

(1) The glossary now includes all network metrics used in the manuscript (betweenness, closeness, density, etc.).

(2) The conclusion now stresses the key differences between publication authorship and Latent Dirichlet Allocation (LDA) in terms of methodology and the clusters found by means of one or the other approach.

Moreover, the journal requirements asked to review the reference list. I can safely say that the references are complete and correct.

With these changes to the manuscript, I hope to have satisfied your critique!
